# Control of Allergic Rhinitis and Asthma Test: A systematic review of measurement properties and COSMIN analysis

**DOI:** 10.1002/clt2.12194

**Published:** 2022-09-25

**Authors:** Rafael José Vieira, Bernardo Sousa‐Pinto, António Cardoso‐Fernandes, Cristina Jácome, Diana Portela, Rita Amaral, Ana Sá‐Sousa, Ana Margarida Pereira, Jean Bousquet, João Almeida Fonseca

**Affiliations:** ^1^ Department of Community Medicine, Information and Health Decision Sciences (MEDCIDS) Faculty of Medicine of the University of Porto Porto Portugal; ^2^ Centre for Health Technology and Services Research Health Research Network (CINTESIS@RISE) Faculty of Medicine of the University of Porto Porto Portugal; ^3^ School of Health Polytechnic of Porto Porto Portugal; ^4^ Department of Women's and Children's Health Uppsala University Uppsala Sweden; ^5^ Allergy Unit CUF Porto Hospital & Institute Porto Portugal; ^6^ Institute for Allergology Charité—Universitätsmedizin Berlin Corporate Member of Freie Universität Berlin and Humboldt‐Universität zu Berlin Berlin Germany; ^7^ Fraunhofer Institute for Translational Medicine and Pharmacology ITMP Allergology and Immunology Berlin Germany; ^8^ University Hospital Montpellier France; ^9^ ARIA Montpellier France

**Keywords:** allergic rhinitis, asthma, CARAT, COSMIN, patient‐reported outcomes

## Abstract

The Control of Allergic Rhinitis and Asthma Test (CARAT) is a patient‐reported outcome measurement (PROM) assessing the control of asthma and allergic rhinitis (AR) at a 4 week interval. This systematic review aimed to evaluate the measurement properties of CARAT. Following PRISMA and COSMIN guidelines, we searched five bibliographic databases and retrieved studies concerning the development, assessment of properties, validation, and/or cultural adaption of CARAT. The studies' methodological quality, the quality of measurement properties, and the overall quality of evidence were assessed. We performed meta‐analysis of CARAT measurement properties. We included 16 studies. Control of Allergic Rhinitis and Asthma Test displayed sufficient content validity and very good consistency (meta‐analytical Cronbach alpha = 0.83; 95% CI = 0.80–0.86;*I* ^2^ = 62.6%). Control of allergic rhinitis and Asthma Test meta‐analytical intraclass correlation coefficient was 0.91 (95% CI = 0.64–0.98;*I* ^2^ = 93.7%). It presented good construct validity, especially for correlations with Patient‐reported outcome measures assessing asthma (absolute Spearman correlation coefficients range = 0.67–0.73; moderate quality of evidence), and good responsiveness. Its minimal important difference is 3.5. Overall, CARAT has good internal consistency, reliability, construct validity and responsiveness, despite the heterogeneous quality of evidence. Control of Allergic Rhinitis and Asthma Test can be used to assess the control of asthma and AR. As first of its kind, this meta‐analysis of CARAT measurement properties sets a stronger level of evidence for asthma and/or AR control questionnaires.

## 1 INTRODUCTION

Patient‐reported outcome measures (PROMs) have been developed to quantify the perceived impact of a specific disease or group of diseases from the patient's perspective.^1^, ^2^ PROMs may provide valuable insights into several disease domains, from the perceived effectiveness of treatments to the quality of life, being crucial to guiding clinical decisions.^3^, ^4^

For assessing the control of asthma or allergic rhinitis (AR), several PROMs are available, including the Asthma Control Test (ACT),^5^ the Asthma Control Questionnaire (ACQ),^6^ the Allergic Rhinitis Control Test (ARCT),^7^ and the Rhinitis Control Assessment Test (RCAT).^8^, ^9^ All of these PROMs assess asthma and AR separately. However, most patients with asthma also have AR^10^, ^11^ and there is a need to simultaneously evaluate these two conditions.^12^ The Allergic Rhinitis and Its Impact on Asthma (ARIA) initiative suggests that both conditions should be holistically evaluated using a single tool.^13^, ^14^ To the best of our knowledge, the Control of Allergic Rhinitis and Asthma Test (CARAT) is the only PROM assessing the control of both asthma and AR (other PROMs developed to be used in patients with asthma and AR either focus on quality of life^15^ or screening of AR in asthmatic patients^16^). It has 10 questions addressing upper and lower airway symptoms, sleep disturbances, limitation of activities, and the need to increase medication in the previous 4 weeks. The total score ranges from 0 to 30 points with scores above 24 points indicating good control of both conditions.^17^

Control of Allergic Rhinitis and Asthma Test development has been thoroughly documented and has been independently assessed by several studies.^18^, ^19^, ^20^ Moreover, CARAT has been widely used in clinical practice and in scientific research, which led to its prompt translation and cross‐cultural adaptation based on international recommendations and best practices.^20^, ^21^, ^22^ It may be administered on paper during medical visits, but it is also available in digital versions, through a website^23^ and mHealth apps for asthma and AR,^24^, ^25^ allowing the patient to use it between clinical assessments.

Hence, the growing use of CARAT prompts the need for a systematic assessment of its measurement (psychometric) properties. Therefore, the purpose of this systematic review was to objectively evaluate the measurement properties of CARAT using the COnsensus‐based Standards for the selection of health status Measurement Instruments (COSMIN) methodology for systematic reviews of PROMs guidelines.^26^

## 2 METHODS

### 2.1 Study design

This systematic review with meta‐analysis was reported according to the recommendations of the Preferred Reporting in the Systematic Reviews and Meta‐Analyses (PRISMA)^27^ and the COSMIN methodology for systematic reviews of PROMs guidelines.^26^ The COSMIN methodology has specific recommendations on the assessment of the risk of bias in primary studies, on the rating of measurement properties and on the assessment of the overall quality of evidence for each measurement property.^26^

### 2.2 Selection criteria

We included original studies (i) assessing adolescents (aged 12 years and older) or adults with asthma and/or AR, and (ii.a) which concerned the development, assessment of properties (such as validity, reliability, consistency and responsiveness), and/or cultural adaption and validation of CARAT or (ii.b) in which such questionnaire was used simultaneously with other PROMs as a study endpoint, and (iii) which used CARAT to assess asthma and/or AR control with a 4‐week recall. We excluded reports available solely as conference abstracts, as recommended by COSMIN.^26^ No restrictions based on publication date or language were applied.

### 2.3 Search strategy

A comprehensive search was performed in January 2022 in five bibliographic databases: Ovid/MEDLINE, Web of Science, Scopus, ClinicalTrials.Gov and the Cochrane Central Register of Controlled Trials (CENTRAL). The detailed search query may be found in Supplementary Table 1. References of the included studies were screened to identify potentially relevant studies. Additionally, we performed a manual search on Google Scholar to identify additional studies that cited any included primary studies on the development, validation or cultural adaptation of CARAT.

### 2.4 Study selection

After eliminating duplicates, two independent authors (RJV and CJ) screened articles' titles and abstracts. The full texts of articles not excluded in the screening phase were independently read by two authors (RJV and ACF). Efforts to contact the investigators were made whenever publications were not accessible by other means. Articles in a language unknown to the reviewers were translated to English either by native speakers of that language or by using an online translator tool.^28^ Any disagreement between the authors was solved by consensus.

### 2.5 Data extraction

The following data were independently extracted from each included primary study by two authors (RJV and ACF) into a purposely built form: sample size, distribution of participants' age and gender, frequency of patients with AR and/or asthma, setting (e.g., primary care, secondary care…), country and language of questionnaire administration. In addition, we retrieved information on the results obtained by each primary study on the measurement properties of CARAT. When more than one report assessed the same participants (or overlapped in the assessed participants), information was retrieved from the article assessing a larger sample, and the remaining articles were screened for additional information not presented in the main article.

### 2.6 Quality assessment

The measurement properties of CARAT were assessed by two independent authors (RJV and ACF) using the COSMIN methodology.^26^ The evaluation of such properties comprised (i) the assessment of the methodological quality of primary studies, (ii) the overall rating of the measurement properties of CARAT, and (iii) the assessment of the generated quality of evidence. The rates applied for each domain are stated in Supplementary Table S2 and further explained below.

The methodological quality of primary studies concerns the risk of bias assessment of the included studies (including those concerning the development of CARAT) regarding each psychometric property on items such as the adoption of the most adequate statistical procedures and measures, sampling and study size. It is rated from ‘very good (V)’ to ‘inadequate (I)’ using the COSMIN risk of bias checklist,^26^ and determined by taking the lowest rating of any items within each measurement property.

The overall rating concerns the quantitative results of each psychometric property, by comparing their quantitative results with pre‐established criteria for good measurement properties (usually predefined cut‐offs). The results for each measurement property of each study were rated qualitatively as ‘sufficient (+)’, ‘insufficient (−)’, or ‘indeterminate (?)’. Content validity was assessed based on the COSMIN methodology recommendations,^29^ using five criteria for relevance, one for comprehensiveness, and four for comprehensibility. For structural validity, a ‘sufficient (+)’ rating required a Root Mean Square Error of Approximation <0.06 or Standardized Root Mean Residuals <0.08. For internal consistency, we required at least low evidence for structural validity and a Cronbach's alpha ≥0.70. Likewise, for reliability, an intraclass correlation coefficient (ICC) of at least 0.70 was required.^26^ For the rating of hypothesis testing for construct validity and responsiveness, we assumed that correlations with instruments (or their changes) should be ≥0.50 when measuring similar constructs and 0.30–0.50 when measuring related, but dissimilar constructs,^30^ or the area under the receiver operating characteristic (ROC) curve should be ≥0.70^26^. The results were then summarized for each measurement property: an overall ‘sufficient’ (+) or ‘insufficient’ (−) rating was given if >75% of results were concurrent, an ‘inconsistent’ (±) rating was given if no rating exceeded 75% and no appropriate explanation for inconsistency could be given, and an ‘indeterminate’ (?) rating was given if all single study results were indeterminate.^26^

Finally, the quality of evidence concerns the confidence in the summarized results based on the Grading of Recommendations Assessment, Development and Evaluation (GRADE) approach. It was rated as high, moderate, low, or very low, taking into account the methodological quality of the studies, the inconsistency of results across studies, imprecision, and indirectness^26^, ^31^

### 2.7 Data analysis

To perform a quantitative synthesis of evidence on the internal consistency, reliability, construct validity and responsiveness of CARAT and its subscales (‘CARAT upper airway’ and ‘CARAT lower airway’), we performed meta‐analyses of Cronbach alphas (internal consistency), intraclass correlation coefficients (ICC; reliability) and Spearman correlation coefficients (construct validity and responsiveness). We were not able to perform a meta‐analysis of other properties (e.g., measurement error), due to insufficient number of included primary studies assessing such properties.

We applied the random‐effects model, using the restricted maximum likelihood method. No primary study presented confidence intervals or standard errors along with their effect size measures. Therefore, for performing meta‐analysis of Spearman correlation coefficients and ICCs, coefficients were firstly transformed according to the formula $0.5\times \ln\left(\frac{1+coefficient\;correlation\;}{1-coefficient\;correlation}\right)$, with their variances being estimated by $\frac{1}{sample\;size-3}$ ^32^; meta‐analytical results were then back‐transformed into the natural scale. For performing meta‐analysis of Cronbach alphas, variances were estimated based on computed confidence interval limits.^33^

Heterogeneity was assessed using the *I* ^2^ statistic and the *p*‐value for the Q‐Cochran statistic—an *I* ^2^>50% and a *p*‐value <0.10 were deemed to represent substantial heterogeneity. Whenever information was available, sensitivity analyses were performed for patients with asthma and patients without asthma. In addition, to ensure inclusion of studies with similar methodology, for outcomes assessed by primary studies using different data retrieving strategies (e.g., outpatient consultation with physicians versus patient self‐reporting through mHealth tools), our main meta‐analytical results were those not considering mHealth data.

All analyses were performed using the metafor package of software *R* (version 4.0).

## 3 RESULTS

### 3.1 Study selection

**FIGURE 1:**
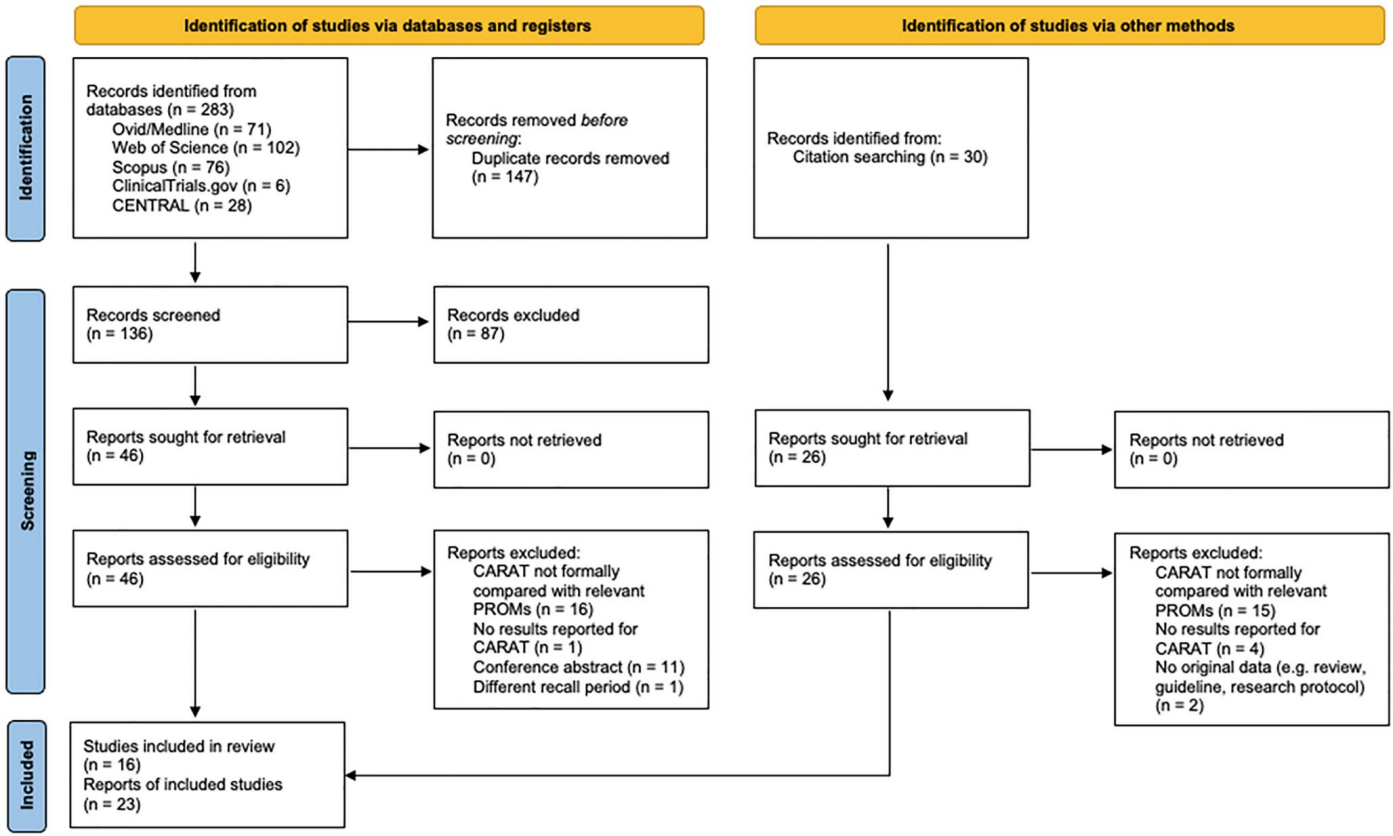
Preferred Reporting Items for Systematic Reviews and Meta Analyses (PRISMA) flow diagram illustrating the studies' selection process

Our database search returned a total of 283 search results (Figure 1). After duplicates removal, a total of 136 references were assessed through title and abstract reading, of which 48 were fully read. We identified and screened 216 unique articles from Google Scholar reference searching, and 30 were fully read. We included 16 original studies (published throughout 23 reports) in our systematic review (Table 1).^17^, ^18^, ^19^, ^20^, ^21^, ^22^, ^34^, ^35^, ^36^, ^37^, ^38^, ^39^, ^40^, ^41^, ^42^, ^43^, ^44^, ^45^, ^46^, ^47^, ^48^, ^49^, ^50^

**TABLE 1:** Characteristics of the included studies (*n* = 4467 participants)

	*n*	Age	Females	Allergic rhinitis *n* (%)	Asthma *n* (%)	Setting	Country	Language
		Mean (SD)	*n* (%)					
Fonseca 2010^35^	193	37.5 (13.84)	131 (67.9)	193 (100)	193 (100)	Secondary care	Portugal	Portuguese
Fonseca 2012^17^	62^a^	39.6 (14.5)	37 (59.7)	62 (100)	62 (100)	Secondary care	Portugal	Portuguese
Lourenço 2014^36^, ^37^	224	46.2^b^	130 (58.0)	224 (100)	120 (53.6)	Pharmacy	Portugal	Portuguese
Sá‐Sousa 2015^38^	364	NR	209 (57.4)	NR	364 (100)	Populational	Portugal	Portuguese
van der Leeuw 2015^18^, ^21^	92^c^	44.0 (13.7)	62 (67.4)	77 (83.7)	52 (56.5)	Primary & secondary care	Netherlands	Dutch
Domingues 2016^19^, ^39^	103	49.5 (18.1)	77 (74.8)	39 (37.9)	103 (100)	Primary care	Portugal	Portuguese
Oudkerk 2016^40^	393	55 (15)	244 (62.1)	153 (38.9)	393 (100)	Diagnostic health care centre	Netherlands	Dutch
Werner 2018^22^	213	50.0 (16.3)	139 (65.3)	101 (47.4)	213 (100)	Secondary care	Germany	German
Gani 2019^41^	113	NR	56 (49.6)	113 (100)	55 (48.7)	Secondary care	Italy	Italian
Pereira Martins 2019^42^	103	NR	39 (37.9)	103 (100)	103 (100)	Secondary care	Portugal	Portuguese
Kosse 2020^43^, ^44^	243	15.1 (2.0)	114 (53.1)	243 (100)	228 (93.8)	Pharmacy	Germany	German
Tosca 2020^45^	50	14.3^d^	15 (30.0)	50 (100)	50 (100)	Secondary care	Italy	Italian
Guedes 2021^46^	105	NR	74 (70.5)	105 (100)	NR	Pharmacy	Portugal	Portuguese
Harbyieli 2021^20^	100^e^	46.6 (13.6)	77 (77.0)	100 (100)	100 (100)	Secondary care	Turkey	Turkish
Jácome 2021^47^	67	NR^f^	NR	NR	67 (100)	Populational	Portugal	Multi‐language
Sousa‐Pinto 2021^48^, ^49^, ^50^	2042	39.0 (12.4)	1507 (73.8)	2042 (100)	1173 (57.4)	Community (mHealth)	25 countries	Multi‐language

Abbreviations: NR, Not reported; SD, Standard deviation.

^a^ 51 patients completely filled out the CARAT10 questionnaire in both visits.

^b^ Standard deviation not reported. Median (IQR) = 48.5 (18–70).

^c^ 44 patients filled in the CARAT10 questionnaire in both the first and second visits.

^d^ Standard deviation not reported.

^e^ 50 patients filled in the CARAT10 questionnaire in both visits.

^f^ Median (IQR) = 20 (17–33).

### 3.2 Characteristics of included studies

Table 1 summarises the characteristics of included studies. The original Portuguese version of CARAT was used in 8 studies (11 reports).^17^, ^19^, ^34^, ^35^, ^36^, ^37^, ^38^, ^39^, ^42^, ^46^, ^47^ There were 2 studies (3 reports) on the Italian,^40^, ^41^, ^45^ German and Dutch^18^, ^21^, ^40^ versions of CARAT.^22^, ^43^, ^44^ The Turkish version was assessed in one study.^20^ One further study, published in 3 reports and using data from a mobile app, enrolled patients from 25 countries, displaying CARAT in multiple languages.^48^, ^49^, ^50^ A total of 4467 participants were assessed, with the mean reported age ranging between 15 and 55 years old. In four studies (eight reports), all the 2622 participants were reported to have AR,^36^, ^37^, ^41^, ^43^, ^44^, ^48^, ^49^, ^50^ while asthma was reported in all the 1245 participants in six studies (seven reports).^19^, ^22^, ^38^, ^39^, ^40^, ^46^, ^47^ Five studies included only patients (*n* = 508) with both AR and asthma.^17^, ^20^, ^35^, ^42^, ^45^

### 3.3 Methodological quality of primary studies

There was variation in the methodological quality ratings for each psychometric property in each individual study, but overall we found a low risk of bias for all assessed psychometric properties (Table 2).

**TABLE 2:** Methodological quality of studies on the measurement properties of the control of allergic rhinitis and asthma test (CARAT)

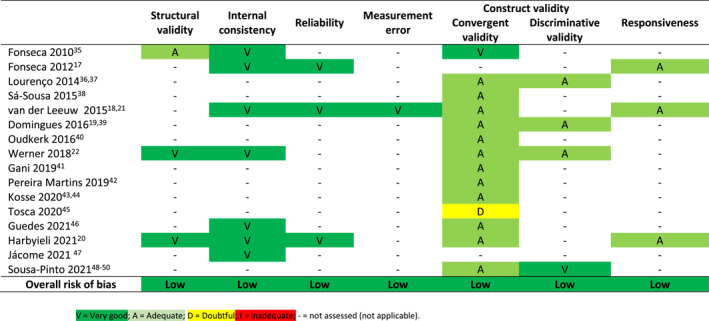

The quality of PROM development for CARAT (Table 3) is rated based on its development study.^34^ The ratings for the general design requirements ranged from ‘adequate’ to ‘very good’. Regarding concept elicitation, although data collection methods were deemed ‘very good’ and a skilled interviewer was used, meetings were recorded but not transcribed verbatim.^34^ The same issue deemed the rating of the assessment of comprehensibility in the pilot test as ‘doubtful’. Comprehensiveness was not assessed in a pilot test, but only in the development process.

**TABLE 3:** Quality of the development of the control of allergic rhinitis and asthma test (CARAT)

Assessed component			Rating
CARAT design	General design requirements	Clear construct	Very good
		Clear origin of construct	Very good
		Clear target population	Very good
		Clear context of use	Very good
		Representativeness of sample	Adequate
	Concept elicitation		Doubtful
	Total CARAT design		Doubtful^a^
Cognitive interview study	Representativeness of sample		Adequate
	Comprehensibility		Doubtful
	Comprehensiveness		Doubtful
	Total cognitive interview study		Doubtful^a^
Total CARAT development			Doubtful^a^

^a^ Based on the lowest rating.

### 3.4 Measurement properties of the CARAT

**FIGURE 2:**
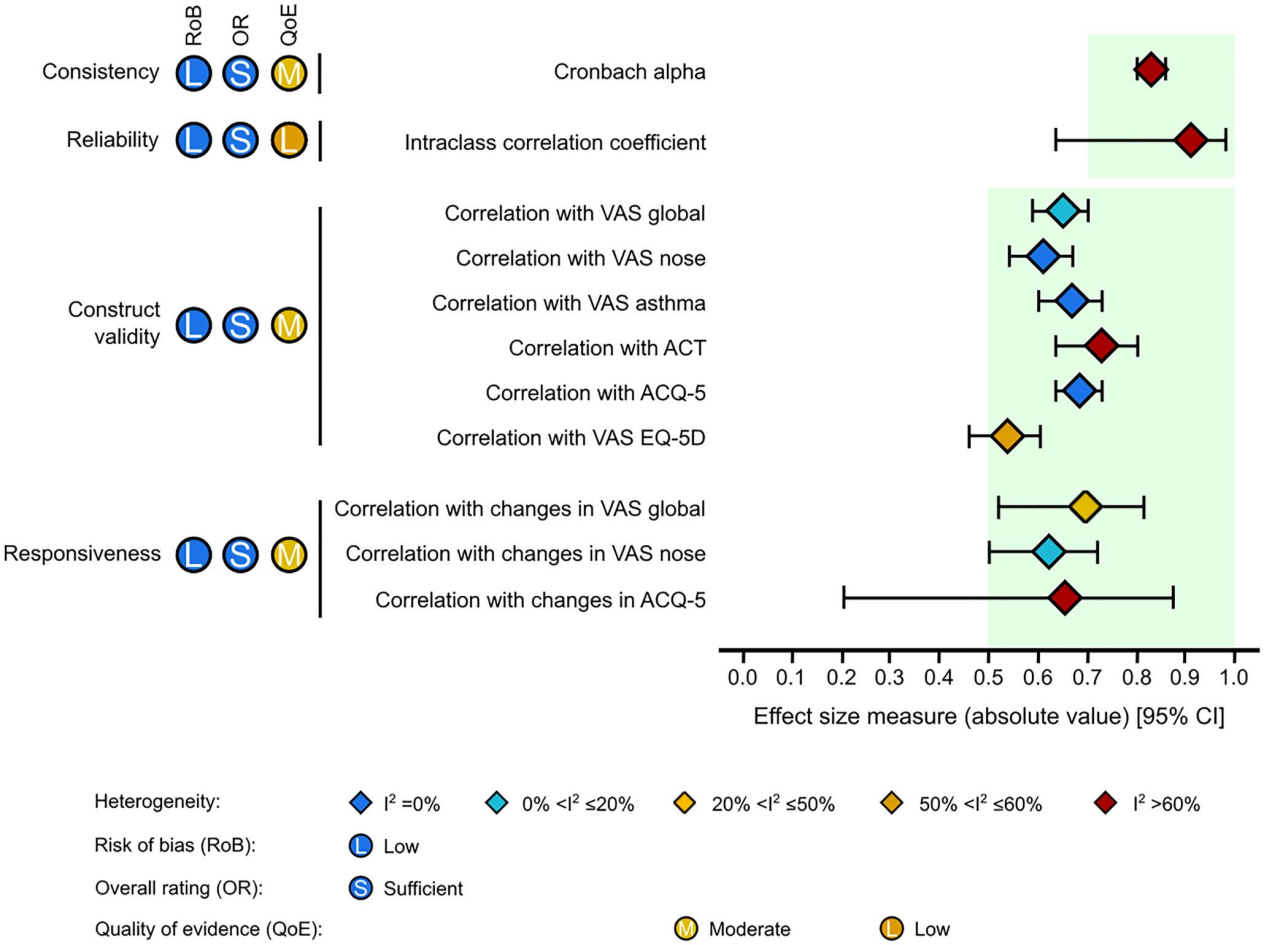
Main meta‐analytical results on the properties of the Control of Allergic Rhinitis and Asthma Test (CARAT). Light green areas indicate the range of good results according to the COSMIN guidelines. ACQ‐5, Asthma Control Questionnaire 5; ACT, Asthma Control Test; CI, Confidence interval; VAS, Visual analogue scale

Supplementary Table S3 and Supplementary Table S4 display the results for the overall rating and quality of evidence assessment for CARAT. Meta‐analytical results are available in detail in Table 4 and Supplementary Table S5, and summarized in Figure 2. It was possible to assess all measurement properties at least once, except for cross‐cultural and criterion validity; regarding the latter, we considered the comparisons between the scores in CARAT and other PROMs as evidence for construct validity (and not of criterion validity), as per the COSMIN guidelines.^26^

**TABLE 4:** Meta‐analytical results for the consistency, reliability, construct validity and responsiveness of the control of allergic rhinitis and asthma test (CARAT)

	*N* primary studies	*N* participants	Meta analytical result (95% CI) [*I* ^2^; *Q*‐Cochran *p*‐value]
Consistency—Cronbach alpha	6	766	0.83 (0.80; 0.86) [62.6%; 0.026]
Reliability—ICC	3	111	0.91 (0.64; 0.98) [93.7%; <0.001]
Construct validity			
Correlation with VAS global^a^	3	509	−0.65 (−0.70; −0.59) [18.7%; 0.311]
Patients with asthma^b^	2	146	−0.56 (−0.66; −0.44) [0%; 0.861]
Patients without asthma	2	865^c^	−0.55 (−0.59; −0.50) [0%; 0.580]
Correlation with VAS nose^d^	3	385	−0.61 (−0.67; −0.54) [0%; 0.788]
Patients with asthma	2	1273^c^	−0.58 (−0.62; −0.54) [0%; 0.969]
Correlation with VAS asthma^e^	2	285	−0.67 (−0.73; −0.60) [0%; 0.334]
Correlation with ACT^f^	4	640	0.73 (0.64; 0.80) [79.2%; 0.001]
Patients with asthma	3	240	0.75 (0.67; 0.81) [34.1%; 0.253]
Patients without asthma	2	176	0.73 (0.31; 0.91) [93.3%; <0.001]
Correlation with ACQ‐5	3	498	−0.68 (−0.73; −0.64) [0%; 0.670]
Correlation with VAS EQ‐5D	2	1492^c^	−0.54 (−0.60; −0.46) [50.7%; 0.155]
Responsiveness			
Correlation with changes in VAS global	2	95	−0.70 (−0.81; −0.52) [47.8%; 0.166]
Correlation with changes in VAS nose	2	151	−0.62 (−0.72; −0.50) [16.9%; 0.273]
Correlation with changes in ACQ‐5	2	95	−0.65 (−0.88; −0.20) [87.5%; 0.005]

Abbreviations: ACQ‐5, Asthma Control Questionnaire 5; ACT, Asthma Control Test; CI, Confidence interval; ICC, Intraclass correlation coefficient; VAS, Visual analogue scale.

^a^ Meta‐analytical results considering also the study of Sousa‐Pinto et al based on MASK‐air^®^ data: −0.62 (95% CI = −0.68; −0.54) [*I* ^2^ = 70.2%; Q‐Cochran *p*‐value = 0.003].

^b^ Meta‐analytical results considering also the study of Sousa‐Pinto et al based on MASK‐air^®^ data: −0.60 (95% CI = −0.63; −0.56) [*I* ^2^ = 0%; Q‐Cochran *p*‐value = 0.793].

^c^ Results including a MASK‐air^®^ data study (Sousa‐Pinto et al, 2021).

^d^ Meta‐analytical results considering also the study of Sousa‐Pinto et al based on MASK‐air^®^ data: −0.57 (95% CI = −0.63; −0.50) [*I* ^2^ = 50.1%; Q‐Cochran *p*‐value = 0.110].

^e^ Meta‐analytical results considering also the study of Sousa‐Pinto et al based on MASK‐air^®^ data: −0.59 (95% CI = −0.72;‐0.42) [*I* ^2^ = 89.7%; Q‐Cochran *p*‐value<0.001].

^f^ Patients without allergic rhinitis: 0.725 (95% CI = 0.308; 0.909) [*I* ^2^ = 93.2%; Q‐Cochran *p*‐value = 0.0001].

#### 3.4.1 Content validity

The assessment of content validity was solely based on the development study^34^ and the authors' opinions (Supplementary Table S3). We rated content validity as ‘sufficient’, albeit with very low quality of evidence, due to the absence of independent individual studies assessing the content validity of CARAT.

#### 3.4.2 Structural validity

Three studies assessed the structural validity of the CARAT,^20^, ^22^, ^35^ which confirmed the two‐factorial scale structure of CARAT.

#### 3.4.3 Internal consistency

The internal consistency of CARAT was assessed in 7 studies (published in 8 reports).^17^, ^18^, ^20^, ^21^, ^22^, ^35^, ^46^, ^47^ Additionally, five of these studies reported on the internal consistency of CARAT subscales.^17^, ^18^, ^20^, ^21^, ^22^, ^35^ The Cronbach alpha was reported as superior to 0.70 in all studies^17^, ^18^, ^20^, ^21^, ^22^, ^35^, ^46^, ^47^ but we qualitatively rated the internal consistency of CARAT and its subscales as ‘indeterminate’ (Table 3), due to absence of enough evidence for sufficient structural validity (as only one study reported on the goodness of fit indices). The quality of evidence on the internal consistency for CARAT was deemed ‘moderate’.

We quantitatively summarized the results for internal consistency (Table 4 and Supplementary Table S5). Overall, CARAT displayed good consistency (meta‐analytical Cronbach alpha = 0.83; 95% CI = 0.80; 0.86), with severe heterogeneity (*I* ^2^ = 62.6%), and similar results were observed for its subscales, although with less heterogeneity (*I* ^2^ = 18.9–45.4%). Heterogeneity was low when assessing solely patients with asthma (i.e., excluding those without a diagnosis of asthma) (meta‐analytical Cronbach alpha = 0.85; 95% CI = 0.83; 0.87; *I* ^2^ = 16.9%).

#### 3.4.4 Reliability

Three studies (published in four reports) assessed the reliability of CARAT.^17^, ^18^, ^20^, ^21^ The reliability was qualitatively rated as ‘adequate’, as all studies resulted in an ICC of 0.70 or higher.^17^, ^18^, ^20^, ^21^ There was heterogeneity in the meta‐analysis results and in the populations included in the underlying studies,^17^, ^18^, ^20^, ^21^ which rendered us to rate the quality of evidence on reliability as ‘low’, despite its adequate ICC.

Meta‐analytical results for reliability show that CARAT displayed high reliability (meta‐analytical ICC = 0.91; 95% CI = 0.64; 0.98), albeit with severe heterogeneity (*I* ^2^ = 93.7%) (Supplementary Table S4).

#### 3.4.5 Measurement error

Measurement error was formally assessed in 2 reports by one study^18^, ^21^ with low risk of bias. The authors found the smallest error of measurement of CARAT to be 2.8 and the minimal clinically important difference to be 3.5.^18^, ^21^

#### 3.4.6 Construct validity

Construct (convergent) validity was assessed in 14 studies (published in 20 reports),^18^, ^19^, ^20^, ^21^, ^22^, ^35^, ^36^, ^37^, ^38^, ^39^, ^40^, ^41^, ^42^, ^43^, ^44^, ^45^, ^46^, ^48^, ^49^, ^50^ but one of them did not report correlation coefficients nor areas under the ROC curve.^45^ For CARAT, 12 studies out of 13 studies (92%) showed ‘sufficient’ evidence for construct validity.^18^, ^19^, ^20^, ^21^, ^22^, ^35^, ^36^, ^37^, ^38^, ^39^, ^40^, ^42^, ^43^, ^44^, ^46^, ^48^, ^49^, ^50^ Eight studies assessed the construct validity for the CARAT subscales^18^, ^19^, ^20^, ^21^, ^22^, ^35^, ^36^, ^37^, ^38^, ^39^, ^48^, ^49^, ^50^ and ‘sufficient’ evidence for construct validity was found in 6 (75%) studies for the upper airway subscale,^18^, ^20^, ^21^, ^22^, ^35^, ^38^, ^48^, ^49^, ^50^ and 7 (87.5%) studies for the lower airway subscale.^18^, ^20^, ^21^, ^22^, ^35^, ^36^, ^37^, ^38^, ^48^, ^49^, ^50^

In quantitative synthesis, CARAT displayed strong correlations with all assessed comparators. Meta‐analytical Spearman coefficients for the correlations between CARAT and daily visual analogue scales (VASs) ranged between −0.61 (95% CI = −0.67; −0.54; *I* ^2^ = 0%) for VAS nose and −0.67 (95% CI = −0.73; −0.60; *I* ^2^ = 0%) for VAS asthma. Higher heterogeneity, however, was observed when considering also the study based on mHealth data.^48^, ^49^, ^50^ For asthma control scores, the meta‐analytical correlation between CARAT and ACT was 0.73 (95% CI = 0.64; 0.80; *I* ^2^ = 79.2%), while that with ACQ‐5 was −0.68 (95% CI = −0.73; −0.64; *I* ^2^ = 0%). A significant but lower correlation was observed for the association between CARAT and EQ‐5D VAS (−0.54; 95% CI = −0.60; −0.46; *I* ^2^ = 50.7%) that assesses the general health state on a specific day.

Considering CARAT subscales, the CARAT upper airway subscale displayed a stronger meta‐analytical correlation with VAS nose and weak‐to‐moderate correlations with VAS asthma and asthma control scores. The reverse was observed for the CARAT lower airway subscale, which displayed strong correlations with VAS asthma and asthma control scores, but weaker correlations with VAS nose (Supplementary Table S5).

#### 3.4.7 Responsiveness

Based on three studies (in four published reports),^17^, ^18^, ^20^, ^21^ we found ‘sufficient’ evidence for the responsiveness of CARAT and its subscales (Table 3). In quantitative synthesis, CARAT displayed high responsiveness regarding all tested outcomes. Meta‐analytical Spearman correlation coefficients ranged between −0.62 (95% CI = −0.72; −0.50; *I* ^2^ = 16.9%) when considering changes in VAS nose to −0.70 (95% CI = −0.81;−0.52; *I* ^2^ = 47.8%) when considering changes in VAS global allergy symptoms.

#### 3.4.8 Cross‐cultural validity

**FIGURE 3:**
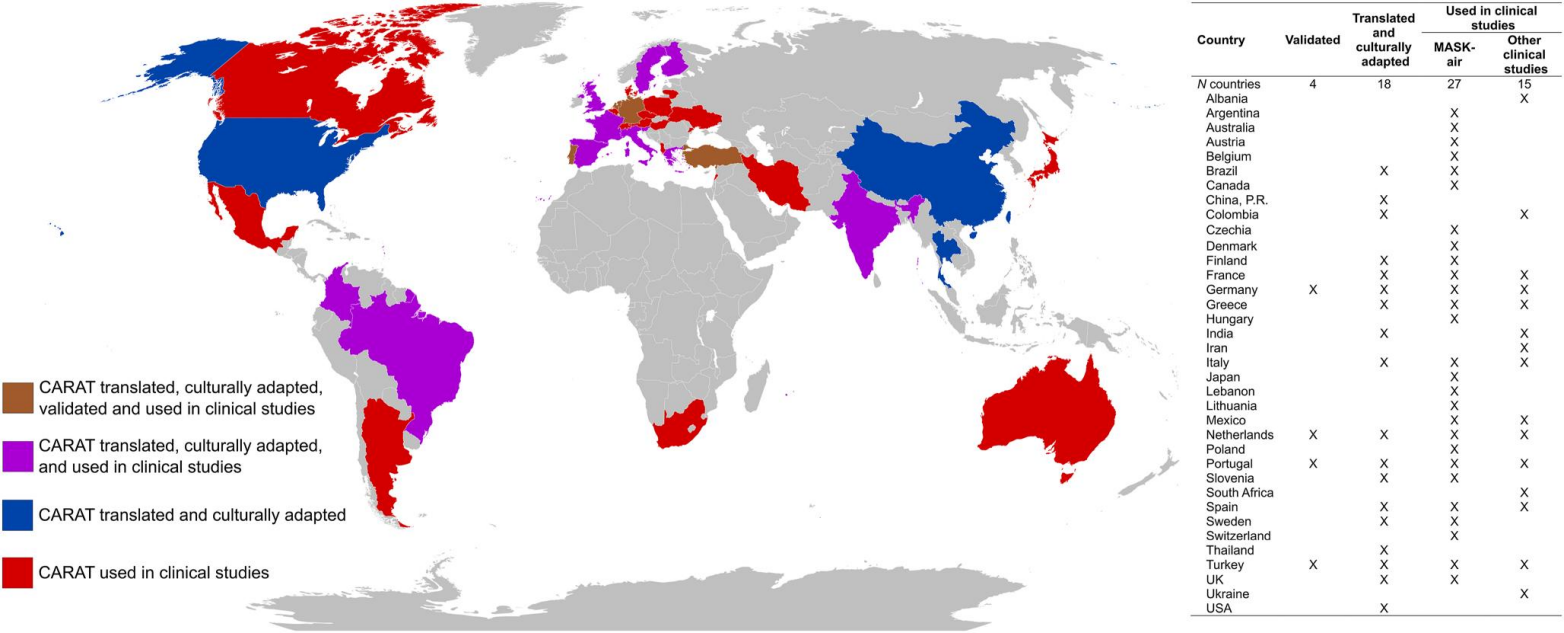
Worldwide availability and use of the Control of Allergic Rhinitis and Asthma Test (CARAT)

No multiple group factor analysis or differential item functioning was performed in cross‐cultural validity studies of CARAT. Nevertheless, we present the measurement properties of CARAT per country reported in validation studies in Supplementary Table S6. The properties are consistent throughout the four countries in which CARAT has been validated with the exception of responsiveness where there is some heterogeneity. Additionally, CARAT has been used in 57 studies of clinical studies from fifteen different countries and is implemented in a mobile app (MASK‐air^®^) currently available in 27 countries (Figure 3).

### 3.5 Interpretability and feasibility

Interpretability of CARAT is summarized in Supplementary Table S7. Overall, the percentage of missing items was low in all studies (between 0% and 9.7%). The percentage of participants reaching the maximum score (ceiling score) ranged between 2.6% and 8.7%. Feasibility is described in Supplementary Table S8. Control of Allergic Rhinitis and Asthma Test is made of 10 questions which take less than three minutes to complete, its use for individual purposes is free and does not require any prior authorization for clinical use.

## 4 DISCUSSION

This is the first systematic review of measurement properties for asthma and/or AR and following the COSMIN guidelines^26^, ^29^ to assess and summarise the psychometric properties of CARAT. Overall, we found that CARAT shows high internal consistency, reliability, construct validity and responsiveness, despite the heterogeneous studies that were included. These results indicate that CARAT can be successfully used to assess the control of asthma and AR with a 4 week recall.

Control of Allergic Rhinitis and Asthma Test was originally developed in Portuguese.^34^, ^35^ Physicians and patients were involved in its development stage. Patient involvement is crucial to ensure that questionnaires include patients' perspectives and are tailored to their needs.^51^ In the development of CARAT, 60 individual interviews were performed by a trained psychologist, but interviews were not transcribed verbatim, leading to a ‘Doubtful’ rating for concept elicitation in the development of CARAT and for the assessment of comprehensibility in the pilot study. However, all other COSMIN recommendations for the development of PROMs were met. Indeed, issues in concept elicitation in PROM development have been reported in other systematic reviews,^1^, ^52^ as PROMs, and in this case CARAT, were developed prior to the publication of COSMIN guidelines.

Content validity refers to whether the content of an instrument appropriately reflects the construct that is being measured and it is often considered the most important measurement property of an instrument.^26^, ^29^ Based on the results from the development study and an independent assessment by two reviewers, we deemed the content validity as ‘sufficient’, as CARAT meets all the topics for relevance and comprehensibility. Comprehensiveness was not assessed in its development study, but CARAT follows the ARIA^14^ and the Global Initiative for Asthma (GINA)^53^ guidelines and the reviewers independently agreed that CARAT includes all the key concepts for the assessment of asthma and AR control. However, there was insufficient evidence on the content validity of CARAT as it was only explored in the development study,^34^ as occurred in systematic reviews of other PROMs. Importantly, the cross‐cultural adaptation of CARAT required that a sample of the target population was enquired on the relevance and comprehensibility of the questionnaire,^12^ but results were not reported (NR) in primary studies. The lack of independent studies assessing the content validity was also a limitation observed in systematic reviews of other PROMs.^1^, ^52^ In fact, it has been previously recognized that content validity has not been rigorously demonstrated for most asthma PROMs.^54^ To the best of our knowledge, there are two published systematic reviews assessing the PROMs commonly used in asthma^13^, ^55^ and one for AR,^56^ which did not evaluate the content validity of the included PROMs. Therefore, our systematic review is the first to systematically assess the content validity of a PROM for asthma and AR.

‘Sufficient’ structural validity is a prerequisite for assessing internal consistency. Three studies confirmed the two‐factorial scale structure of CARAT.^20^, ^22^, ^35^ It was not possible to determine the quality of structural validity for CARAT, since the goodness of fit indices were reported in only one study.^22^ Nevertheless, performing meta‐analyses on the Cronbach's alpha (internal consistency) demonstrated the good internal consistency of CARAT. The COSMIN guidelines recommend the risk of bias to be increased when studies do not report the Cronbach's alpha for its subscales, which was the case for two of the studies included.^46^, ^47^ However, CARAT was developed as a global instrument to assess both asthma and AR control, following ARIA's vision that asthma and AR are interdependent conditions which should be managed simultaneously.^14^ In addition, its subscales have not been validated independently and are not recommended for widespread use. Consequently, although we present the synthetized evidence for CARAT subscales in this systematic review, we opted not to increase the risk of bias on the internal consistency of CARAT.

There is no comparable gold standard assessing asthma and AR control. As a result, we considered the comparisons between CARAT and other validated PROMs to be evidence for construct validity (namely convergent validity), as per the COSMIN guidelines.^26^ We found good correlations with low heterogeneity between the CARAT score and other PROMs, namely the VASs and ACQ‐5. Likewise, we found a good correlation for the comparison between the CARAT score and the ACT, but with substantial heterogeneity, which can be partly explained by the inclusion of patients with AR without asthma in the primary studies (which were not so present in studies assessing correlation with ACQ‐5). Indeed, when performing sensitivity analysis and quantitatively pooling the results only from studies including patients with asthma, we found that the heterogeneity was greatly decreased. Regarding the lower correlation with the EQ‐5D VAS, it is important to note that CARAT and EQ‐5D measure related but dissimilar constructs, and that EQ‐5D may not be the best quality of life measure to be used in asthma^57^ as (i) it does not react very sensitively to small changes in asthma control,^58^ (ii) VAS EQ‐5D is less sensitive than ACQ‐6 to assess asthma control,^59^ and (iii) it incompletely represents the deficits of quality of life in severe asthma.^60^ Therefore, CARAT shows good correlation with asthma PROMs, even in patients without AR. Importantly, we included only studies assessing the original recall period of 4 weeks. One study validated CARAT to be used with a 1 week recall period.^25^ Although not included in this systematic review, its results are consistent with those described here.

Control of Allergic Rhinitis and Asthma Test displayed good reliability and responsiveness, albeit with some heterogeneity. Regarding cross‐cultural validity, it is important to note that CARAT has been translated, culturally adapted and clinically validated for German,^22^, ^43^, ^44^ Dutch^18^, ^21^ and Turkish^20^ following a protocol which was based on international standards,^12^ but no results on measurement invariance were reported, precluding the assessment of cross‐cultural validity. Nevertheless, we found consistent results reported in validation studies of CARAT performed in different countries. Additionally, it has been translated and culturally adapted for 27 other languages,^61^ used in clinical research in 15 different countries and is currently integrated into a mHealth app with users from 27 different countries.

Comparing our results with those from the ACT and the ACQ development/validation studies^5^, ^6^, ^62^ (Supplementary Table S9), CARAT displays similar internal consistency to ACT (CARAT: 0.83; ACT: 0.84–0.85; NR in the original ACQ development or validation studies) and similar to higher reliability (CARAT: 0.91; ACT: 0.77; ACQ: 0.90). Comparisons regarding construct validity and responsiveness are limited by the fact that the ACT and the ACQ used different PROMs as references in their original development or validation studies^5^, ^6^, ^62^ The correlations between CARAT, ACT and ACQ scores and clinician impression of disease control show ACQ to have the strongest correlation (0.67), followed by CARAT (0.57) and ACT (0.45–0.52). On the other hand, CARAT displays a higher area under the ROC curve (0.82)^35^ compared to ACT (0.77) (NR in ACQ).^5^, ^6^, ^62^

This review has some limitations, mostly stemming from the included primary studies. In fact, as previously reported, relevant information was often missing from primary studies as CARAT was developed prior to the publishing of COSMIN guidelines, sometimes leading to evidence downgrading or to the impossibility of performing meta‐analysis, as commonly found in systematic reviews of PROMs. An additional limitation consists of the diversity of assessed populations and data collection methods (including based on mHealth). We tried to overcome this limitation by performing separate meta‐analysis for different patient subgroups (e.g., AR without asthma, asthma, asthma without AR), but separate information on the properties of CARAT was not always available for each subgroup. Future studies should, therefore, present results in more detail, particularly on the internal consistency and reliability when asthma or AR are the sole diagnoses, and convergent validity and responsiveness for patients with AR only. Additionally, there is a lack of studies comparing CARAT and questionnaires for AR, precluding the assessment of the performance of CARAT in patients with AR without asthma. Importantly, the small number of included primary studies precluded us from following other approaches for identification of sources of heterogeneity (e.g., meta‐regression). Additionally, there is insufficient information on the underlying quality of evidence of other PROMs used in asthma, including on their development and content validity, as previously noted.^54^ Therefore, there is a need for further systematic reviews with meta‐analysis on other PROMs used in asthma and/or AR.

This study has also important strengths. Although previous studies performed systematic reviews of PROMs used in asthma^13^ and AR,^56^ they did not pool quantitative evidence by meta‐analysis and did not follow the COSMIN methodology recommendations. One additional systematic review performed meta‐analysis on the accuracy of the ACT and the ACQ,^55^ but it does not follow the COSMIN recommendations and takes a diagnostic performance approach assessing only sensitivity, specificity, likelihood ratios, diagnostic odds ratio and area under the ROC curve. Therefore, our study is the first systematic review to follow the COSMIN guidelines and to qualitatively and quantitatively assess the measurement properities of a PROM used in asthma or AR, thereby setting a stronger level of evidence for asthma and/or AR control questionnaires. The obtained evidence supports the use of CARAT to assess the control of asthma and AR in clinical practice. Another strength of our study is the inclusion in the literature search of cross‐referencing using Google Scholar, in order not to miss any relevant publications meeting the inclusion criteria of our review. Additionally, two independent authors were involved in all steps of this review, which was especially relevant for assessing the content validity of CARAT. We were able to perform meta‐analyses on several properties of the questionnaire, thus better summarizing the evidence on the measurement properties of CARAT. Importantly, especially for convergent validity, heterogeneity was, overall, low.

In conclusion, this systematic review with meta‐analysis summarises for the first time, both qualitatively and quantitatively, the measurement properties of a control questionnaire for asthma or AR. We observed moderate quality evidence for construct validity and responsiveness of CARAT. We also report high internal consistency and reliability, although this is based on lower quality of evidence, mostly reflecting heterogeneity in the underlying primary studies. These results indicate that CARAT can be successfully used to assess the control of asthma and AR with a 4‐week recall. Still, more research is needed on the use of CARAT in patients diagnosed solely with asthma or AR. We also identified the need for synthesis research on the measurement properties of other PROMs available for asthma and AR.

## AUTHOR CONTRIBUTIONS

Rafael José Vieira participated in data extraction, evidence analysis and manuscript writing; Bernardo Sousa‐Pinto participated in conceptualization, evidence synthesis and manuscript writing; António Cardoso‐Fernandes participated in data extraction and evidence analysis; Cristina Jácome participated in conceptualization, data extraction and critical review of the manuscript; Diana Portela participated in manuscript writing; Rita Amaral and Ana Sá‐Sousa participated in conceptualization and critical review of the manuscript; Ana Margarida Pereira participated in manuscript writing and critical review of the manuscript; Jean Bousquet and João Almeida Fonseca participated in conceptualization, manuscript writing and critical review of the manuscript.

## CONFLICT OF INTEREST

João Almeida Fonseca, Cristina Jácome, Rita Amaral, Ana Sá‐Sousa, Ana Margarida Pereira and Jean Bousquet were involved in the original studies of the development, validation and/or mobile phone adaptation of CARAT. Jean Bousquet has developed the MASK‐air^®^ app, in which the CARAT questionnaire is included.

## Supporting information

Supporting Information S1
