# Supplementary material for: Control of Allergic Rhinitis and Asthma Test: A systematic review of measurement properties and COSMIN analysis
Source: Clin Transl Allergy. 2022 Sep 25;12(9):e12194. doi: 10.1002/clt2.12194 (PMC9510765; doi:10.1002/clt2.12194)
Supplement: Supplementary file 1 — Supporting Information S1 [file CLT2-12-e12194-s001.docx]

**Supplementary Table 1. List of queries used for searching electronic databases.**

| **Number** | **Query** | **Search results** |
| --- | --- | --- |
| **Medline via OVID** | | |
| #1 | exp Asthma/ | 135440 |
| #2 | (asthma$ or asthmat$).mp. | 191361 |
| #3 | Bronchial Hyperreactivity/ | 7486 |
| #4 | exp Bronchoconstriction/ | 4247 |
| #5 | Bronchial Spasm/ | 4335 |
| #6 | bronchospas$.mp. | 5618 |
| #7 | (bronch$ adj3 spas$).mp | 4695 |
| #8 | bronchoconstrict$.mp. | 11725 |
| #9 | (bronch$ adj3 constrict$).mp. | 739 |
| #10 | ((bronchial$ or respiratory or airway$ or lung$) adj3 (hypersensitiv$ or hyperreactiv$ or allerg$ or insufficiency)).mp. | 70122 |
| #11 | ((dust or mite$) adj3 (allerg$ or hypersensitiv$)).mp. | 5676 |
| #12 | wheez$.mp. | 14773 |
| #13 | 1 or 2 or 3 or 4 or 5 or 6 or 7 or 8 or 9 or 10 or 11 or 12 | 256295 |
| #14 | exp Rhinitis/ | 36573 |
| #15 | (rhinit$ or rhinoconjunctivit$).mp. | 48770 |
| #16 | exp Allergic Rhinitis/ | 22580 |
| #17 | (hayfever or "hay fever" or pollenosis or pollinosis or SAR).mp. | 26672 |
| #18 | 14 or 15 or 16 or 17 | 70510 |
| #19 | (CARAT or CARAT?10).mp. | 148 |
| #20 | (control$ adj2 allerg$ adj2 rhinit$ adj2 asthm$).mp. | 61 |
| #21 | 19 or 20 | 181 |
| #22 | (13 or 18) and 21 | 71 |
| **ISI Web of Science** | | |
| #1 | (TS=(asthma* OR asthmatic*) OR TS=(bronchospasm*) OR TS=(bronch* NEAR/3 spasm*) OR TS=(bronchoconstrict*) OR TS=(bronch* NEAR/3 constrict*) OR TS=((bronchial* or respiratory or airway* or lung*) NEAR/3 (hypersensitiv* or hyperreactiv* or allerg* or insufficiency)) OR TS=((dust or mite*) NEAR/3 (allerg* or hypersensitiv*)) OR TS=(wheez*)) | 245219 |
| #2 | (TS=(rhinit* or rhinoconjunctivit*) OR TS=(hayfever or "hay fever" or pollenosis or pollinosis or SAR)) | 121222 |
| #3 | (TS=(CARAT or CARAT$10) OR TS=(control* NEAR/2 allerg* NEAR/2 rhinit* NEAR/2 asthm*)) | 560 |
| #4 | (#1 OR #2) AND #3 | 102 |
| **Scopus** | | |
| #1 | ( TITLE-ABS-KEY(asthma* OR asthmatic*)  OR TITLE-ABS-KEY(bronchospas*)  OR TITLE-ABS-KEY(bronch* pre/3 spas*)  OR TITLE-ABS-KEY(bronchoconstrict*)  OR TITLE-ABS-KEY(bronch* pre/3 constrict*)  OR TITLE-ABS-KEY((bronchial* or respiratory or airway* or lung*) pre/3 (hypersensitiv* or hyperreactiv* or allerg* or insufficiency))  OR TITLE-ABS-KEY((dust or mite*) pre/3 (allerg* or hypersensitiv*))  OR TITLE-ABS-KEY(wheez*))  OR ( TITLE-ABS-KEY(rhinit* or rhinoconjunctivit*)  OR TITLE-ABS-KEY(hayfever or "hay fever" or pollenosis or pollinosis or SAR))  AND ( TITLE-ABS-KEY(CARAT or CARAT10 or CARAT-10)  OR TITLE-ABS-KEY(control* pre/2 allerg* pre/2 rhinit* pre/2 asthm*) ) | 76 |
| **ClinicalTrials.gov** | | |
| #1 | CARAT OR CARAT10 OR CARAT-10\| EXPANSION[Concept]Asthma OR EXPANSION[Concept]Rhinitis | 6 |
| **Cochrane Central Register of Controlled Trials (CENTRAL)** | | |
| #1 | MeSH descriptor: [Asthma] explode all trees | 12067 |
| #2 | (asthma* or asthmat*) | 38070 |
| #3 | MeSH descriptor: [Bronchial Hyperreactivity] this term only | 582 |
| #4 | MeSH descriptor: [Bronchoconstriction] explode all trees | 569 |
| #5 | MeSH descriptor: [Bronchial Spasm] this term only | 393 |
| #6 | Bronchospas* | 2169 |
| #7 | bronch* NEAR/3 spas* | 554 |
| #8 | Bronchoconstrict* | 2676 |
| #9 | bronch* NEAR/3 constrict* | 233 |
| #10 | (bronchial* or respiratory or airway* or lung*) NEAR/3 (hypersensitiv* or hyperreactiv* or allerg* or insufficiency) | 5961 |
| #11 | (dust or mite*) NEAR/3 (allerg* or hypersensitiv*) | 1268 |
| #12 | wheez* | 3151 |
| #13 | #1 or #2 or #3 or #4 or #5 or #6 or #7 or #8 or #9 or #10 or #11 or #12 | 44465 |
| #14 | MeSH descriptor: [Rhinitis] explode all trees | 4060 |
| #15 | rhinit* or rhinoconjunctivit* | 11308 |
| #16 | MeSH descriptor: [Rhinitis, Allergic] explode all trees | 3183 |
| #17 | hayfever or "hay fever" or pollenosis or pollinosis or SAR | 2498 |
| #18 | #14 or #15 or #16 or #17 | 12534 |
| #19 | CARAT or CARAT10 or CARAT-10 | 46 |
| #20 | control* NEAR/2 allerg* NEAR/2 rhinit* NEAR/2 asthm* | 18 |
| #21 | #19 or #20 | 58 |
| #22 | (#13 or #18) and #21 | 28 |

**Supplementary Table 2. Ratings used to assess the methodological quality of primary studies (including studies on PROM development), the overall rating of measurement properties, and the quality of available evidence, according to the COSMIN guidelines.**

| **Methodological quality^a^** | **Overall rating of measurement properties** | **Quality of evidence** |
| --- | --- | --- |
| Very good (V) | Sufficient (+) | High |
| Adequate (A) | Inconsistent (±) | Moderate |
| Doubtful (D) | Indeterminate (?) | Low |
| Inadequate (I) | Insufficient (-) | Very low |

^a^ Risk of bias of primary studies, including PROM development.

**Supplementary Table 3. Content validity of the Control of Allergic Rhinitis and Asthma Test (CARAT).**

|  | **Development study** | **Rating of reviewers** | **Overall rating** | **Quality of evidence** |
| --- | --- | --- | --- | --- |
| **Relevance** |  | | **+** | Very low |
| Are the included items relevant for the construct of interest? | **+** | **+** |  |  |
| Are the included items relevant for the target population of interest? | **+** | **+** |  |  |
| Are the included items relevant for the context of use of interest? | **+** | **+** |  |  |
| Are the response options appropriate? | **+** | **+** |  |  |
| Is the recall period appropriate? | **+** | **+** |  |  |
| **Comprehensiveness** |  |  | **±** | Very low |
| Are all key concepts included? | **-** | **+** |  |  |
| **Comprehensibility** |  |  | **+** | Very low |
| Are the PROM instructions understood by the population of interest as intended? | **+** |  |  |  |
| Are the PROM items and response options understood by the population of interest as intended? | **+** |  |  |  |
| Are the PROM items appropriately worded? |  | **+** |  |  |
| Do the response options match the question? |  | **+** |  |  |
| **Content validity** |  |  | **+** | **Very low** |

+ = sufficient; – = insufficient; ± = inconsistent; ? = indeterminate.

**Supplementary Table 4.** **Overall qualitative rating and quality of evidence for measurement properties of the Control of Allergic Rhinitis and Asthma Test (CARAT) and its upper airway (CARAT-UA) and lower airway (CARAT-LA) subscales.**

|  | **CARAT** | | **CARAT-UA** | | **CARAT-LA** | |
| --- | --- | --- | --- | --- | --- | --- |
|  | **Overall rating** | **Quality of evidence** | **Overall rating** | **Quality of evidence** | **Overall rating** | **Quality of evidence** |
| **Structural validity** | ? | Moderate | NA | NA | NA | NA |
| **Internal consistency** | ? | Moderate | ? | Moderate | ? | Moderate |
| **Reliability** | + | Low | ?^a^ | NA | ?^a^ | NA |
| **Measurement error** | + | Moderate | ?^a^ | NA | ?^a^ | NA |
| **Construct validity** | + | Moderate | + | Moderate | + | Moderate |
| **Responsiveness** | + | Moderate | + | Moderate | + | Moderate |

+ = sufficient; – = insufficient; ± = inconsistent; ? = indeterminate; NA = not applicable. ^a^ = Rated “Indeterminate”, following COSMIN guidelines, due to results not being reported.

**Supplementary Table 5. Meta-analytical results for the consistency, reliability, construct validity and responsiveness of the Control of Allergic Rhinitis and Asthma Test (CARAT) upper airway and lower airway subscales.**

|  | ***N* primary studies** | ***N* participants** | **Meta analytical result (95%CI) [*I*^2^; Q-Cochran *p*-value]** |
| --- | --- | --- | --- |
| **A. CARAT upper airway subscale** | | | |
| Consistency - Cronbach alpha | 5 | 661 | 0.81 (0.77;0.84) [45.4%; 0.127] |
| Construct validity |  |  |  |
| Correlation with VAS global ^a^ | 2 | 285 | -0.56 (-0.70;-0.39) [70.2%;0.067] |
| Correlation with VAS nose ^b^ | 3 | 385 | -0.74 (-0.82;-0.62) [75.8%; 0.016] |
| Correlation with VAS asthma ^c^ | 3 | 509 | -0.46 (-0.52;-0.39) [0%; 0.737] |
| Correlation with ACT | 2 | 313 | 0.29 (0.16;0.41) [28.1%; 0.238] |
| Patients with asthma | 2 | 201 | 0.37 (0.24;0.48) [0%; 0.938] |
| Correlation with ACQ-5 | 3 | 498 | -0.36 (-0.43;-0.28) [0%; 0.581] |
| Responsiveness |  |  |  |
| Correlation with changes in VAS nose | 3 | 195 | -0.72 (-0.84;-0.53) [77.7%; 0.009] |
| **B. CARAT lower airway subscale** | | | |
| Consistency - Cronbach alpha | 5 | 661 | 0.84 (0.82;0.86) [18.9%; 0.186] |
| Construct validity |  |  |  |
| Correlation with VAS global ^d^ | 2 | 285 | -0.64 (-0.70;-0.57) [0%; 0.410] |
| Correlation with VAS nose ^e^ | 3 | 385 | -0.39 (-0.48;-0.29) [17.1%; 0.276] |
| Correlation with VAS asthma ^f^ | 3 | 509 | -0.67 (-0.73;-0.62) [0%; 0.613] |
| Correlation with ACT | 3 | 537 | 0.82 (0.76;0.86) [64.1%; 0.077] |
| Patients with asthma | 2 | 201 | 0.86 (0.81;0.89) [0%; 0.427] |
| Correlation with ACQ-5 | 3 | 498 | -0.78 (-0.83;-0.72) [55.7%; 0.111] |
| Responsiveness |  |  |  |
| Correlation with changes in VAS nose | 2 | 151 | -0.37 (-0.57;-0.13) [57.0%; 0.127] |
| Correlation with changes in VAS asthma | 2 | 95 | -0.60 (-0.80;-0.29) [73.4%; 0.053] |
| Correlation with changes in ACQ-5 | 2 | 95 | -0.63 (-0.87;-0.15) [88.4%; 0.003] |

ACQ-5=Asthma Control Questionnaire 5; ACT=Asthma Control Test; CI=Confidence interval; ICC=Intraclass correlation coefficient; VAS=Visual analogue scale

^a^ Meta-analytical results considering also the study of Sousa-Pinto et al based on MASK-air^®^ data: -0.54 (95%CI=-0.63;-0.44) [*I*^2^=71.6%; Q-Cochran *p*-value=0.030]; ^b^ Meta-analytical results considering also the study of Sousa-Pinto et al based on MASK-air^®^ data: -0.69 (95%CI=-0.79;-0.55) [*I*^2^=92.0%; Q-Cochran *p*-value<0.001]; ^c^ Meta-analytical results considering also the study of Sousa-Pinto et al based on MASK-air^®^ data: -0.37 (95%CI=-0.52;-0.18) [*I*^2^=91.3%; Q-Cochran *p*-value<0.001]; ^d^ Meta-analytical results considering also the study of Sousa-Pinto et al based on MASK-air^®^ data: -0.58 (95%CI=-0.70;-0.42) [*I*^2^=89.0%; Q-Cochran *p*-value<0.001]; ^b^ Meta-analytical results considering also the study of Sousa-Pinto et al based on MASK-air^®^ data: -0.39 (95%CI=-0.42;-0.36) [*I*^2^=0.1%; Q-Cochran *p*-value=0.462]; ^c^ Meta-analytical results considering also the study of Sousa-Pinto et al based on MASK-air^®^ data: -0.64 (95%CI=-0.69;-0.58) [*I*^2^=51.0%; Q-Cochran *p*-value=0.10

**Supplementary Table 6. Measurement properties reported in cross-cultural validation studies of CARAT.**

|  | **Population** | **Internal consistency**^a^ | **Reliability**^b^ | **Convergent validity**^c^ | | | | |  | **Responsiveness**^c^ |  |
| --- | --- | --- | --- | --- | --- | --- | --- | --- | --- | --- | --- |
|  |  |  |  | **VAS Global** | **VAS Nose** | **VAS Asthma** | **ACQ-5** | **ACT** | **VAS Global** | **VAS Nose** | **ACQ-5** |
| Portugal^17,35^ | Asthma + AR | 0.85 | 0.82 | -0.69 | -0.61 | -0.69 | -0.71 | NR | -0.76 | -0.69 | -0.79 |
| Germany^22^ | Asthma ± AR | 0.87 | NR | NR | NR | NR | -0.66 | 0.60 | NR | NR | NR |
| Netherlands^18,21^ | AR ± Asthma | 0.82 | 0.80 | -0.69 | -0.64 | -0.62 | -0.67 | NR | -0.61 | NR | -0.45 |
| Turkey^20^ | Asthma + AR | 0.83 | 0.98 | NR | -0.58 | NR | NR | 0.80 | NR | -0.58 | NR |

^a^ Cronbach's alpha. ^b^ Intracluster correlation coefficient. ^c^ Spearman correlation coeficiente. NR = Not reported. VAS = Visual Analogue Scale. ACQ-5 = Asthma Control Questionnaire-5. ACT = Asthma Control Test.

**Supplementary Table 7. Interpretability of the Control of Allergic Rhinitis and Asthma Test (CARAT) and its upper airway (CARAT-UA) and lower airway (CARAT-LA) subscales.**

|  |  | **Percentage of missing items or of missing total scores** | **Floor scores (%)^a^** | **Ceiling scores (%)^b^** | **Subgroups data** | **MIC/MID** | **Completion time** |
| --- | --- | --- | --- | --- | --- | --- | --- |
| Fonseca 2010 | CARAT | Information for the 16 item version. Percentage of missing items ranging from 0 to 2.1% (nasal pruritus and throat symptoms) | 0.00 | 2.59 | Gender; Age; asthma and rhinitis severity as per physician classification | – | – |
|  | CARAT-LA |  | – | – | – | – | – |
|  | CARAT-UA |  | – | – | – | – | – |
| Fonseca 2012 | CARAT | Percentage of missing items ranged from 0 to 9.7%. Only for one item the percentage of missing items was higher than 3.2% | 0.00 | 4.84 | Patients' self-perceived control | – | – |
|  | CARAT-LA | Percentage of missing items ranged from 0 to 9.7%. Only for one item the percentage of missing items was higher than 3.2% | 0.00 | 29.03 | Patients' self-perceived control | – | – |
|  | CARAT-UA | Percentage of missing items ranged from 0 to 3.2% | 8.06 | 6.45 | Patients' self-perceived control | – | – |
| Domingues 2015 | CARAT | – | – | – | Asthma with and without allergic rhinitis | – | – |
|  | CARAT-LA | – | – | – | – | – | – |
|  | CARAT-UA | – | – | – | – | – | – |
| van der Leeuw 2015 | CARAT | – | 0.00 | 8.70 | Gender; Age | 3.5 | – |
|  | CARAT-LA | – | – | – | – | – | – |
|  | CARAT-UA | – | – | – | – | – | – |
| Werner 2018 | CARAT | 8% questionnaires had at least one item missing - 4% had one item missing; 4% had more than one item missing and were excluded. Among included questionnaires, % of missing items ranged from 0.5 to 1.4% | – | – | Allergic rhinitis vs no allergic rhinitis | – | – |
|  | CARAT-LA | Among included questionnaires, % of missing items ranged from 0.5 to 1.4% | – | – | Allergic rhinitis vs no allergic rhinitis | – | – |
|  | CARAT-UA | Among included questionnaires, % of missing items ranged from 0.9 to 1.4% | – | – | Allergic rhinitis vs no allergic rhinitis | – | – |
| Harbiyeli 2021 | CARAT | – | 0.00 | – | Gender, education status, employment status, smoking status, presence of polyps, multiple allergen sensitivity, | – | – |
|  | CARAT-LA | – | – | – | Gender, education status, employment status, smoking status, presence of polyps, multiple allergen sensitivity, | – | – |
|  | CARAT-UA | – | – | – | Gender, education status, employment status, smoking status, presence of polyps, multiple allergen sensitivity, | – | – |

a = Percentage of users obtaining the minimum score in the questionnaire. b = Percentage of users obtaining the maximum score in the questionnaire

**Supplementary Table 8. Feasibility of the Control of Allergic Rhinitis and Asthma Test (CARAT) questionnaire.**

| Patients' comprehensibility | Adolescents and adults |
| --- | --- |
| Clinicians' comprehensibility | Any clinician |
| Type and ease of administration | Paper or online (computer or mobile) |
| Length of the instrument | 10 questions |
| Completion time | < 3 minutes |
| Patients' required mental and physical ability level | No required specific physical ability. Basic literacy |
| Ease of score calculation | Easy (sum of points for each question) |
| Copyright | This questionnaire is copyrighted mostly to prevent unapproved changes to it. Its use for individual purposes (e.g. supporting clinical assessment at a patient consultation) is free and does not requires any autorization. The use of the questionnaire by any research group, to aggregate data from different patients, requires a communication to the CARAT group. For-profit organizations or the use of the questionnaire with commercial/marketing purpose require case-by-case authorization from the CARAT group. |
| Cost of an instrument | None for clinical care and academic studies (may have costs for other uses) |
| Required equipment | None other than paper and pen OR computer OR smartphone |
| Availability in different settings | Available in any setting (clinical setting or home) |
| Regulatory agency's requirement for approval | Not required |

**Supplementary Table 9. Comparison between the Control of Allergic Rhinitis and Asthma Test (CARAT), the Asthma Control Test (ACT) and the Asthma Control Questionnaire (ACQ) measurement properties. Results for ACT and ACQ are extracted from the corresponding original development and validation studies.**

| **Measurement property** | **CARAT** | **ACT** | **ACQ** |
| --- | --- | --- | --- |
| Internal consistency (Cronbach alpha (95% CI)) | 0.83 (0.80;0.86)^a^ | 0.84 – 0.85^b,c^ | NR^d^ |
| Reliability (ICC (95% CI)) | 0.91 (0.64;0.98)^a^ | 0.77 ^c^ | 0.90 ^d^ |
| Correlation with clinician impression | 0.57^e,f,g^ | 0.45 – 0.52^b,c,h^ | 0.67 ^d,h^ |
| AUROC (reference: clinical impression) | 0.82^e,i^ | NR ^c^ | 0.77 ^d^ |

ACQ — Asthma Control Questionnaire. ACT — Asthma Control test. AUROC — Area Under the Receiver Operating Characteristics Curve. CARAT — Control of Allergic Rhinitis and Asthma Test. ICC — Intraclass Correlation Coefficient. NR — Not reported.

^a^ — obtained from this study. ^b^ — range. ^c^ — obtained from the original development/validation studies.^5,62^ ^d^ —obtained from the original development/validation studies.^6^ ^e^ — obtained from the original validation study.^35^ ^f^ — Spearman correlation coefficient. ^g^ — Clinical impression for asthma control only. For allergic rhinitis, correlation is 0.52. ^h^ — Pearson correlation coefficient. i — AUROC for overall allergic rhinitis and asthma control; AUROC for allergic rhinitis is 0.80 and AUROC for asthma is 0.82
